# Flavor Characterization of Traditional Fermented Soybean Pastes from Northeast China and Korea

**DOI:** 10.3390/foods12173294

**Published:** 2023-09-01

**Authors:** Shanshan Zhao, Yuhang Sai, Wanting Liu, Huiwen Zhao, Xue Bai, Wanying Song, Yan Zheng, Xiqing Yue

**Affiliations:** 1 College of Food Science, Shenyang Agricultural University, Shenyang 110866, China; 2 Shenyang Key Laboratory of Animal Product Processing, Shenyang Agricultural University, Shenyang 110866, China

**Keywords:** soybean paste, electronic tongue, SPME-GC-MS, volatile compounds, chemometrics

## Abstract

This study compares the physicochemical properties, taste, and volatile compounds of Northeastern Chinese dajiang (C) and Korean doenjang (K) and distinguishes the discriminant volatile metabolites between them. The result revealed that compared to group C, group K exhibited more similar physicochemical properties and had lower pH, moisture, and amino acid nitrogen content, while demonstrating higher titratable acidity, salt content, and reduced sugar content. The electronic tongue analysis showed that the saltiness and umami of soybean pastes had high response values, enabling clear differentiation of the overall taste between the two types of soybean pastes. A total of 71 volatile substances from the soybean pastes were identified through solid-phase microextraction gas chromatography–mass spectrometry. Furthermore, orthogonal partial least squares discriminant analysis revealed 19 volatile compounds as differentially flavored metabolites. Our study provides a basis for explaining the differences in flavor difference of Northeastern Chinese dajiang and Korean doenjang from the perspective of volatile metabolites.

## 1. Introduction

**Figure 1:**
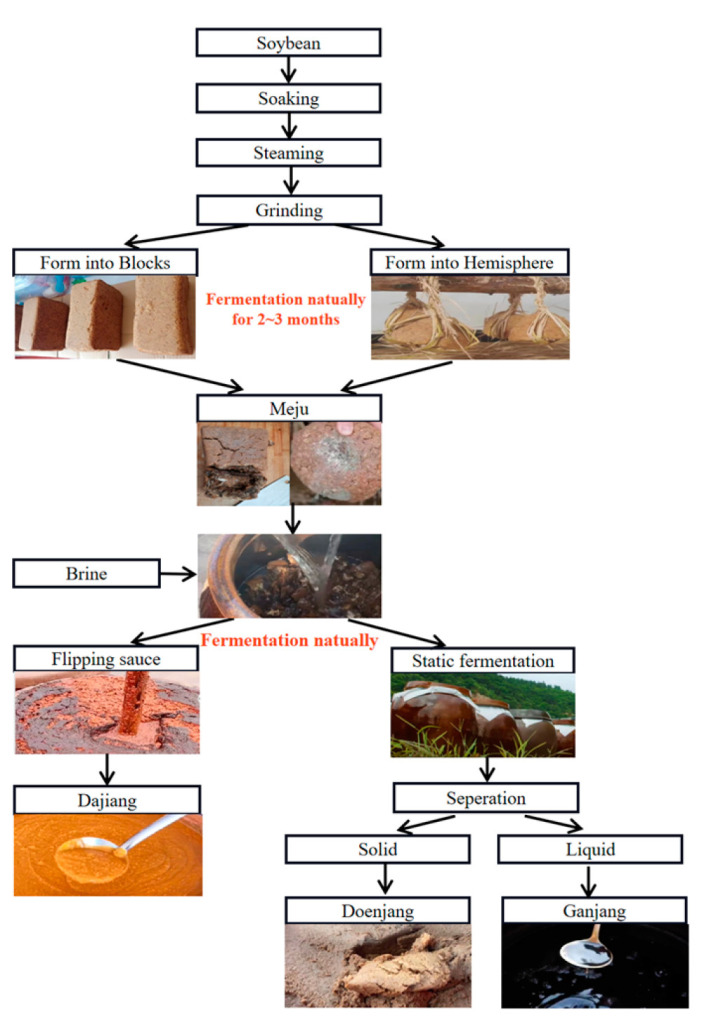
The production process of Chinese dajiang and Korean doenjang.

The fermented soybean paste, which is known for its unique flavor and rich nutritional values, is a widely consumed condiment in East Asia [1,2]. Fermentation enhances the bioavailability of vitamins, proteins, isoflavones, and minerals and reduces the anti-nutritional substances in soybeans [3]. Moreover, this process contributes to various beneficial properties such as antioxidant [4], antimutagenic [5], antibacterial [6], anti-inflammatory [7], anticancer [8], and immunoregulatory properties [9]. Owing to variations in raw materials, production processes, and environments, soybean pastes from different countries and regions have distinct flavors. Dajiang, doenjang, and miso are representative fermented soybean products originating from China, Korea, and Japan, respectively, each characterized by their distinctive flavors [10]. Miso is commonly fermented through inoculation with a mold (such as *Asperigillus orgzae*) [11], whereas dajiang and doenjang are both produced from soybeans through natural fermentation [12,13]. The production process of both dajiang and doenjang consists of two fermentation stages: meju-making and sauce-making. The meju-making process for both types of soybean pastes is similar. It involves soaking, steaming, grinding, shaping, and natural fermentation (as shown in Figure 1). However, there are distinct steps that set apart the production of Northeast Chinese dajiang and Korean doenjang. In the case of dajiang, flipping the sauce is a crucial step, whereas for doenjang, solid–liquid separation plays a unique role in the process. The oxygen introduced during the flipping of the soybean paste encourages the growth of certain aerobic bacteria, which may contribute to the different flavors observed between the two types of soybean pastes. Notably, recent studies have compared the primary and secondary metabolites and volatile compounds, for several fermented soy products, including meju, doenjang, and cheonggukjang [14]. The results showed that most volatile compounds were more abundant in doenjang, whereas the contents of pyrazines, maltol, methoxyphenol, and 3-methylbutanoic acid were higher in cheonggukjang. However, flavors between dajiang and doenjang, which undergo similar fermentation processes, have not been directly compared.

Flavors, including volatile compounds (odors) and nonvolatile components (tastes), are produced by microorganisms and enzymes that degrade sugars, proteins, and fats present in raw materials [15,16,17]. The flavor of soybean pastes is a crucial property that affects consumer acceptability [18]. Taste-related substances, such as amino acids, soluble sugars, and inorganic salts, are the main sources of umami, sweetness, sourness, and saltiness in soybean pastes [19,20]. The taste of soybean pastes is a composite effect arising from these taste-related components. Similarly, the formation of soybean paste odor is a collective result of various volatile components, especially aroma-active compounds. Flavor-related components play a critical role for consumers in the assessment of sensory quality and overall acceptability of the fermented soybean paste [21]. Therefore, quantifying certain flavor-related substances is essential for understanding the causes of flavor variations, establishing correlations between flavor and sensory properties, and evaluating the flavor quality of soybean pastes. The electronic tongue (e-tongue) provides information on molecules or compounds that contribute to the taste of a sample. The e-tongue has recently been applied in many food fields, such as quality identification, and geographical traceability, yielding satisfactory results [19,22,23]. Gas chromatography–mass spectrometry (GC-MS) is a widely employed technique for detecting volatile compounds. It combines the strong separation capability of gas chromatography with the precise identification capacity of mass spectrometry. Unlike other detectors, mass spectrometers offer heightened sensitivity, superior selectivity, and the advantage of delivering structural insights into the component. While single quadrupole mass spectrometers exhibit somewhat diminished quantitative and qualitative capabilities compared to triple quadrupole mass spectrometers, they remain extensively used for volatile compound detection in diverse food types, when coupled with gas chromatography. This adoption is due to their ability to fulfill the demands of routine detection purposes [12,24,25]. In recent years, HS-SPME-GC-MS combined with chemometrics has emerged as a key tool for distinguishing different food flavors and selecting different metabolites from different foods [26,27,28]. Lu et al. [29] effectively distinguished traditional Chinese fermented shrimp pastes from different regions by combining HS-SPME-GC-MS and chemometric analyses. Moreover, Zhao et al. [30] successfully selected 16 and 22 differential metabolites from raw and cooked rice in different regions of China, respectively, by combining HS-SPME-GC-MS and chemometrics. Hexanal, 3,5-octadien-2-one, and 2-butyl-2-octenal were identified as discriminant marks common to both raw and cooked rice.

Therefore, this study aims to (i) compare the physicochemical, taste, and volatile flavor compounds of Northeastern Chinese dajiang and Korean doenjang and (ii) select the differential volatile metabolites present in two types of soybean pastes. This study can provide some information for distinguishing the flavor differences in Northeastern Chinese dajiang and Korean doenjang from the perspective of volatile metabolites.

## 2. Materials and Methods

### 2.1. Sample Collection

A total of 14 samples of traditional fermented soybean paste were purchased from different markets. Seven samples of Northeastern Chinese dajiang (group C) were collected from the Liaoning province, while representative samples of Korean doenjang (group K) were collected from Yanbian Korean Autonomous Prefecture. All the collected samples were produced by the farmers following the process in Figure 1 and were naturally fermented for around 7 months. They were stored at 4 °C until they were analyzed. The detailed information on samples was listed in Table 1.

### 2.2. Physicochemical Characteristics Analysis

Color parameters were analyzed using a Chroma-meter CR-400 (Konica Minolta, Shanghai, China), and the lightness (L*), redness (a*), and yellowness (b*) values were recorded. The moisture contents were detected by drying at 105 °C [31]. Prior to the determination of pH, titratable acidity (TA), salt, reducing sugar, and amino acid nitrogen content, the soybean paste samples were pretreated using the same method. In total, 5 g of a soybean paste sample was mixed with 45 mL distilled water and homogenized on a rotating incubator at 100 rpm, 30 °C for 0.5 h. Then, the soybean paste samples were centrifuged at 5000 rpm for 10 min, and the supernatant was collected and filtered. Subsequently, the pH was measured by a pH meter (leici, Shanghai, China), and the TA was measured by titration with sodium hydroxide (0.1 M) according to the method suggested by Zhang et al. [32]. The content of NaCl was measured according to the method suggested by Yu et al. [19]. A mixture of 100 mL of distilled water, 1 mL of potassium chloride (50 g/L), and 2 mL of sample filtrate was titrated with 0.1 M silver nitrate until it appeared reddish-orange, and the volume of silver nitrate consumed was recorded. At the same time, distilled water was used as a blank experiment. The content of sodium chloride was calculated according to Equation (1).

(1) $$X=\frac{\left(V1-V0\right)\times c\times 0.0585}{10\times 2/100}\times 100$$

*X* is the NaCl content of soybean paste, g/100 g; *V*1 and *V*0 are the volume of silver nitrate consumed by the sample dilution and reagent blank, respectively, mL; *c* is the concentration of silver nitrate standard solution, M; and 0.0585 is the mass of sodium chloride equivalent to 1.00 mL of standard silver nitrate solution (*c*(AgNO_3_) = 1.000 M), g.

Reducing the sugar content was measured by the 3,5-dinitrosalicylic acid (DNS) method [33]. Mixtures of different concentrations of glucose solution with DNS solution were configured as Supplementary Table S1. The mixture was boiled in a water bath for 5 min, cooled with cold water, fixed to 10 mL, and finally, its absorbance was measured at 540 nm. The standard curve was plotted based on the absorbance of different concentrations of glucose standard solution. The absorbance of the sample filtrate was determined using the same method and the content of reducing sugar in the sample was calculated according to the standard curve. The formaldehyde titration method was utilized to test the content of amino acid nitrogen [33]. A mixture of 10 mL of the sample filter with 60 mL of distilled water was titrated to pH 8.2 with 0.05 M NaOH solution. In total, 10 mL of formaldehyde was added to the mixture of pH 8.2, and then it continued to be titrated to a pH of 9.2 with 0.05 M NaOH, and the volume of NaOH solution consumed was recorded. A blank experiment was carried out with distilled water, and the amino acid nitrogen content was calculated according to Equation (2).

(2) $$X=\frac{\left(V1-V0\right)\times c\times 0.014}{m\times V2/V3}\times 100$$

*X* is the amino acid nitrogen content of soybean paste, g/100 g; *V*1 and *V*0 are the volume of NaOH solution consumed in the titration of the sample solution and the blank experiment, respectively, mL; *V*0 is the volume of NaOH solution consumed in the blank experiment, mL; *c* is the concentration of NaOH standard solution, mol/L; 0.014 represents the mass of nitrogen equivalent to 1.00 mL of NaOH standard solution (c(NaOH) = 1.000 M)), g; *m* is the mass of the sample, g; and *V*2 and *V*3 are the amount and the constant volume of the diluted sample filter, respectively, mL.

### 2.3. E-Tongue Analysis

E-tongue analysis was conducted using a PEN3 E-TONGUE (SA402B, Insent company, Houki City, Kanagawa Prefecuture, Japan). The instrument contains five electrodes, namely CT0, AEI, AAE, C00 and CA0, respectively. These five electrodes are attached with specific materials that could respond to different tastes. In total, 10 g of milled soybean paste was weighed and concentrated to 100 mL with distilled water and homogenized on a rotating incubator at 100 rpm, 30 °C for 0.5 h. Then, the soybean paste samples were centrifuged at 5000 rpm for 10 min, and the supernatant was collected and filtered. The clarified sample extraction solution was equally poured into the small beaker equipped with the e-tongue system for testing. The sourness, bitterness, umami, astringency, saltiness, richness, aftertaste astringency, and aftertaste bitterness were tested. The detection parameters were set as follows: cleaning of electrodes for 339 s, equilibration for 30 s, sample measurement for 30 s, and aftertaste measurement for 30 s. Each sample was tested four times and the last three measurements were selected for further analysis.

### 2.4. Volatile Compounds Analysis

The volatile compounds were analyzed according to Jia et al. [34] methods with minor changes. A headspace solid-phase micro-extraction (HS-SPME) was used to extract volatile compounds. In total, 2 g of soybean paste, 2 g of sodium chloride, 6 mL of distilled water, and 5 μL of 2-octanol (25 mg/L, internal standard) were added into a sealed dedicated bottle containing a small rotator. Then, the bottle was preheated at 55 °C water bath for 5 min with a stirring speed of 55 r/min, and the volatile compounds were adsorbed using the DVB/car/pdmsspme fiber (2 cm, 50/30 μm) (Supelco, Bellefonte, Pennsylvania, USA) for 45 min under the same condition. Subsequently, the concentrated volatile compounds were desorbed for 5 min using gas chromatography–mass spectrometry (GC-MS) (7890A, 5975C, Agilent Technologies, Santa Clara, California, USA) with a DB-5MS column (60 m × 0.25 mm × 0.25 µm) for volatile compounds separation. The chromatographic conditions were as follows: injection port temperature 250 °C, flow rate 1 mL/min, splitless mode, and carrier gas He. The initial temperature of the GC oven was set to 32 °C, increased to 180 °C at 5 °C/min and held for 5 min, then ramped up to 200 °C at 5 °C/min, and maintained for 8 min. Finally, the temperature was increased to 230 °C at 5 °C/min, which was maintained for 10 min. The MS condition was as follows: MS ion source temperature 230 °C, electron energy 70 eV, and scanning mass range of 33–450 amu. The volatile compounds preliminary identification was performed by comparing the mass spectra of all metabolites with those in the NIST11.0 library, with a match of at least 80%. Retention indices were compared with reference values for further identification of volatile compounds. A semiquantitative analysis was conducted on the basis of the internal standard 2-octanol. The retention indices (RI) and volatile compounds content were calculated as shown in Equations (3) and (4).

(3) $$RI=\frac{\left(Tn+1-Tx\right)\left(Tx-Tn\right)}{Tn+1-Tn}\times 100\%+100n$$

(4) $$C=\frac{Ax\times C0\times V}{A0\times m\times 1000}$$

*Tn* and *Tn* + 1 are the retention time of n-alkanes before and after the flow to be measured, min; Tx is the retention time of the object to be measured, min; *n* is the number of carbon atoms contained in the previous n-alkane in the stream to be tested; *c* is the volatile compound content, μg/kg; *Ax* and *A0* are the peak area of the volatile compounds and the internal standard, respectively, AU·min; *C*0 represents the mass concentration of internal standard, μg/μL; *V* represents the volume of internal standard, μL; and *m* represents the quantity of soybean paste sample, g.

### 2.5. Statistical Analysis

All measurements were performed three times. All data were presented as mean value ± standard deviation (SD, *n* = 3) and analyzed by SPSS 26.0 (SPSS Inc., Chicago, IL, USA) using a one-way analysis of variance and Duncan’s multiple range test. Principle component analysis (PCA) was performed with Origin 2022 (Origin Lab Corporation, Northampton, Massachusetts, USA). Clustering heat map analysis and Orthogonal Partial Least Squares-Discriminant Analysis (OPLS-DA) were carried out using the Metware Cloud, a free online platform for data analysis (https://cloud.metware.cn (accessed on 9 August 2023). The volatile compounds with VIP > 1.0 and *p* < 0.05 were selected as differential volatile metabolites.

## 3. Results and Discussion

### 3.1. Color Properties of Soybean Paste Samples

The color of soybean pastes is a highly intuitive indicator of its desirability to consumers. The L*, a*, and b* values of soybean pastes between dajiang and doenjang are shown in Table 2. The L*, a*, and b* values of the soybean paste samples ranged from 32.26 to 40.34, 1.82 to 5.39, and 10.05 to 17.08, respectively. Compared to group K, the group C samples had a wider range of L* and b* values. The a* and b* values of all samples were higher than 0, and the a* values of the group C sample were lower than those of the group K sample. This indicates that the color of the soybean paste samples tends to be reddish-yellow and the color of the group K sample tends to be more red. Among the soybean paste samples, C3 had the lowest L*(32.26), a*(1.82), and b*(10.05) values, whereas C5 had the highest L* and b* values. The formation of soybean paste color is closely related to the Maillard reaction [35,36]. Reducing sugar, as the substrate of the Maillard reaction, affects the color and flavor of soybean paste [18].

### 3.2. pH, Titratable Acidity (TA), Moisture, Salinity, Reducing Sugar, and Amino Acid Nitrogen Content of Soybean Paste

**Figure 2:**
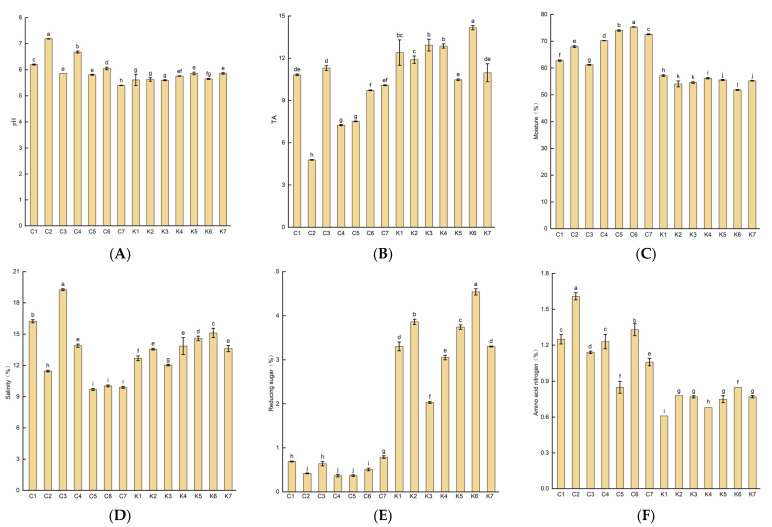
Physicochemical properties of soybean pastes from Northeastern China and Korea. (**A**) pH, (**B**) titratable acid (TA), (**C**) moisture; (**D**) salinity, (**E**) reducing sugar, (**F**) amino acid nitrogen. Group C represents Northeastern Chinese dajang; Group K represents Korean doenjang. Data are presented as mean standard error from triplicates. Different letters indicate significant differences at *p* < 0.05.

In this study, the pH, TA, salinity, moisture, reducing sugar, and amino acid nitrogen concentrations of the soybean pastes were analyzed (Figure 2). The pH and TA values of all soybean paste samples ranged from 5.6 to 7.2 and from 4.8 to 14.2, respectively. Korean doenjang had a lower pH (5.6–5.9) and higher TA (10.97–14.17) compared to those of Northeastern Chinese dajiang. During the fermentation of the soybean paste, microorganisms (such as lactic acid bacteria and *Bacillus*) can utilize the sugars in the raw materials to produce organic acids (such as lactic and acetic acids), thereby reducing the pH and increasing the TA of the soybean pastes [15,37]. Therefore, the doenjang samples exhibit a lower pH and higher TA, which may be attributed to the differences in the fermentation process or raw material composition.

The moisture and salt content results for the soybean paste samples are shown in Figure 2C, D. The moisture concentration in all of the soybean paste samples ranged from 51.8% to 74.7%, with an average moisture content of 62.1%. Compared with group K, group C had a higher moisture concentration (61.2%–74.0%). This is due to the fact that the solid–liquid separation step was conducted during the preparation of Korean doenjang. No significant differences were observed in mean salt concentrations between dajiang and doenjang. Among all the soybean paste samples, C3 and C5 had the highest and lowest salt concentrations of 19.3% and 9.7%, respectively. The salt content can increase the saltiness and umami of the soybean paste and prevent rotting at high salt levels. However, excessive salt content can be harmful to human health [38,39,40]. The reducing sugar concentrations of the soybean pastes are shown in Figure 2E. The reduced sugar concentration of the soybean pastes ranged from 0.4% to 4.5%, and the average content of reducing sugar in group K was 6.28 times higher than that in group C. This may be attributed to the special step (solid–liquid separation), which was performed during the production process of doenjang. Kim et al. [41] and Byeon et al. [42] found that the contents of reduced sugar in Korean doenjang (solid) and Ganjang (liquid) ranged from 1.12 to 14.19 g/100 g and from 0.12 to 1.46 g/100 mL, respectively, with a mean value of 4.93 g/100 g and 0.72 g/100 mL. Reducing sugar serves as a carbon source for microbial growth during the fermentation of the soybean paste and participates in the Mallard reaction to form the color and harmonize the taste of the soybean paste. The amino acid nitrogen content of the soybean paste is an important indicator to evaluate the quality of the soybean paste, which reflects the maturity and quality of the soybean paste. Figure 2F shows the analysis results of the amino acid nitrogen content of the soybean paste. The concentrations of amino acid nitrogen in the soybean paste samples ranged from 0.6% to 1.6%, with an average value of 1.0%. Compared to group C, the amino acid nitrogen content of group K was more similar, ranging from 0.6% to 0.9%. The average contents of amino acid nitrogen in dajiang and doenjang were 1.21 g/100 g and 0.73 g/100 g, respectively. The amino acid nitrogen content was lower in doenjang than that in dajiang; the result was consistent with that reported in previous studies [41,43].

### 3.3. E-Tongue Analysis

**Figure 3:**
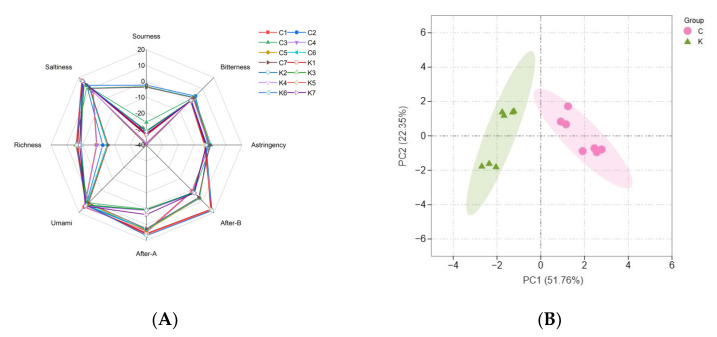
(**A**) Radar chart of the taste responses of dajiang and doenjang samples, and (**B**) principal component analysis (PCA) of E−tongue data. Group C represents Northeastern Chinese dajang; Group K represents Korean doenjang.

The e-tongue analysis is considered an effective tool for analyzing the overall taste because of its ability to rapidly determine the ease of handling and high sensitivity. As shown in Figure 3A, the e-tongue analysis showed a strong response to saltiness and umami, indicating that the soybean paste samples were rich in substances that exhibit both saltiness and umami, such as salt, amino acids, and flavorful peptides. Significant differences were observed in the sourness and richness between the two groups; group C had a higher sourness and lower richness than group K. This difference in acidity is attributed to the differences in the types and contents of organic acids produced by microbial metabolism, such as lactic and acetic acids [37]. Research has shown that lactic acid is the most abundant organic acid in soybean paste [12].

Principal component analysis (PCA) can be used to distinguish the similarities and differences between different samples. The results of the PCA of the e-tongue analysis are shown in Figure 3B. The first two principal components explained 74.11% of the total variance contribution rate, and the samples were clearly divided into two groups, indicating that there were differences in the overall taste of the two groups of soybean pastes.

### 3.4. Comparison of Volatile Compound Profiles for Soybean Pastes

**Figure 4:**
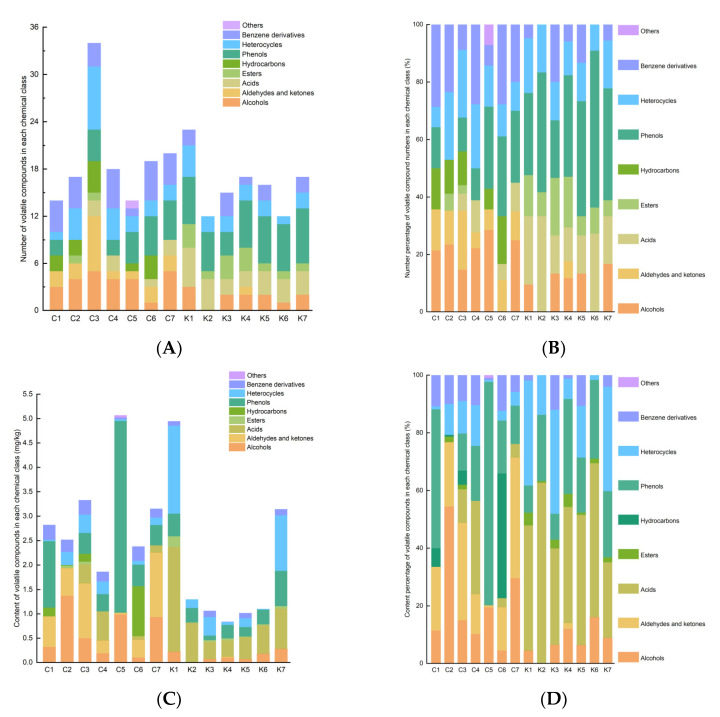
Numbers and contents of volatile substances (**A**,**C**), relative percentages of quantity and content of volatile compounds (**B**,**D**) in different soybean paste samples. Group C represents Northeastern Chinese dajang; Group K represents Korean doenjang.

Volatile compounds in the soybean pastes from Northeast China and Korea were detected using HS-SPME/GC-MS. A total of 71 volatile compounds were identified, including 9 alcohols, 8 aldehydes and ketones, 9 acids, 5 esters, 6 hydrocarbons, 8 phenols, 15 heterocycles, 10 benzene derivatives, and 1 other volatile substance (Table S2). The numbers and contents of the volatile compounds are shown in Figure 3 and Table S3. Compared to group K, the soybean paste samples from group C exhibited a richer variety of and possessed a higher content of volatile compounds. Phenols (19%–50%) were the most diverse categories in Northeastern Chinese dajiang and Korean doenjang (Figure 4). No hydrocarbons were detected in Korean doenjang, and only one type of aldehydes (α-ethylidene-benzeneacetaldehyde) was found. Samples C3 and K6 were identified as the samples with the highest and lowest number of volatile component species, with 40 and 12 species, respectively. In addition, compared to Chinese dajiang, Korean doenjang contains more abundant acid compounds. Northeastern Chinese dajiang had a higher percentage of alcoholic compounds than Korean doenjang, particularly in the C5 sample (28.6%). Korean doenjang had a higher percentage of acids (11.8%–33.3%) and a smaller percentage of aldehydes and ketones (0%–5.9%), in contrast to Northeastern Chinese dajiang. Particularly, K2 and K7 contained a higher content percentage of phenols with 41.7% and 41.2%, respectively. In addition, heterocyclic compounds were found in all soybean paste samples, and the average levels of heterocyclic compounds in seven doenjang and seven dajiang samples were 573.13 μg/kg and 171.62 μg/kg, respectively.

**Figure 5:**
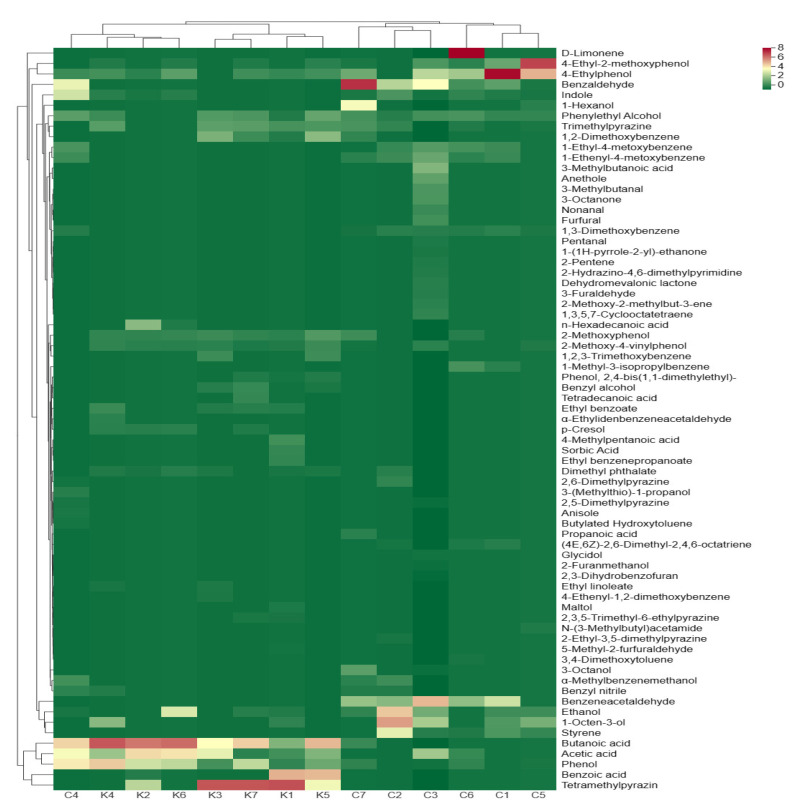
A clustered heat map analysis based on volatile compounds of soybean paste samples. The heat map is visualized after Z scores normalization for volatile compounds content; red represents high content and green represents low content; the names of volatile compounds are listed on the right side of the heat map.

We performed a cluster analysis on the volatile compounds in the soybean pastes to visualize the content of each volatile compound and the similarity of volatile profiles in the soybean paste samples (Figure 5). Fourteen soybean paste samples were clustered into two categories, with six dajiang samples clustered into one category, and C4 clustered with seven doenjang samples. These indicate that the volatile profiles in C4 are more similar to those of seven doenjang samples, while the volatile profiles in dajiang excluding C4 are more similar. Alcohols contribute to both odor and taste and are precursors of ester components in the soybean paste, such as ethanol, phenylethyl alcohol, 1-octen-3-ol, and 3-octanol. Ethanol and 1-octen-3-ol are considered important alcohols in soybean paste because of their abundance. 1-Octen-3-ol had a relatively high concentration in C2 and C5, imparting a mushroom-like odor to soybean paste (Figure 5). Aldehydes and ketones commonly have fruity and malt aromas [44]; furthermore, compared to those in Korean doenjang, more aldehydes and ketones, such as benzaldehyde and benzeneacetaldehyde, have been found in Northeastern Chinese dajiang. Phenols are considered to contribute to the smoky attributes of the soybean paste and are mainly generated by the conversion of ferulic acid through Candida yeast [45]. Many esters have a fruity odor, which can mask the negative flavor of fermented foods [46]. Multiple ethyl compounds (such as ethyl benzoate, ethyl benzenepropanoate, and ethyl linoleate) have been identified by HS-SPME-GC-MS. Various ethyl esters can affect the flavor of the final product. Various benzenes have also been identified in the soybean paste, which is usually produced via the shikimate and benzoic acid pathways [47,48]. Some benzenoid compounds, such as benzyl nitrile, have aromatic properties, which can affect the flavor of the soybean paste through roasted bread, moss, and rose odors. Owing to their high threshold, hydrocarbons usually have a small impact on the overall flavor of the product, whereas heterocyclics contribute significantly to the flavor of the product because of their lower threshold. The heterocyclic components in the soybean paste are mainly pyrazines and furans, which confer the soybean paste with nutty and caramel aromas, respectively [1,12]. In addition, other heterocyclic components (such as 2-hydrazino-4,6-dimethylpyrimidine, anethole) have been found only in the C3 sample.

### 3.5. Distribution of Differential Flavor Compounds in Soybean Paste Samples

**Figure 6:**
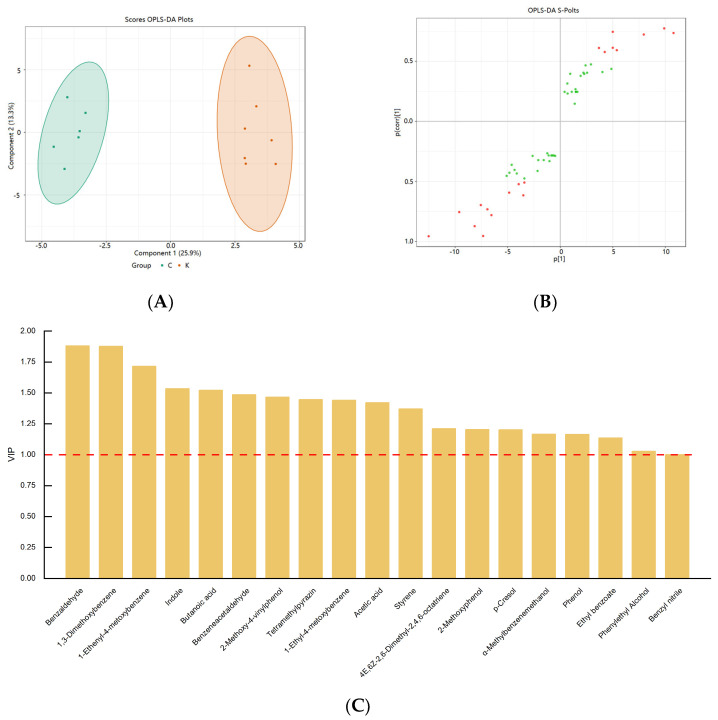
Orthogonal partial least squares discriminant analysis (OPLS−DA) score plot (**A**) and S−plot (**B**) of soybean paste samples from Northeast China and Korea. (R^2^Y = 0.984, Q^2^ = 0.845). VIP values for volatile compounds. Discriminatory volatile markers in soybean paste samples from Northeast China and Korea (**C**) (VIP > 1, *p* < 0.05).

Orthogonal partial least squares discriminant analysis (OPLS−DA) is a supervised analysis method that maximizes differences between groups by combining orthogonal signals and partial least squares discriminant analysis (PLS−DA) to screen the significantly discriminant metabolites. The most significant metabolites were selected using a variable importance projection (VIP). The results of the OPLS−DA analysis are shown in Figure 6. The score plot (Figure 6A) shows well-separated clusters of the soybean paste samples (R^2^Y = 0.984), which represents the goodness of prediction (Q^2^ = 0.845). In our study, the R^2^Y and Q^2^ values of OPLS−DA were acceptable, indicating that Northeastern Chinese dajiang and Korean doenjang could be clearly distinguished.

In this study, 19 volatile metabolites with VIP values greater than 1 were analyzed, including 2 acids, 2 alcohols, 4 phenols, 1 ester, 2 aldehydes, 2 hydrocarbons, 2 heterocycles, and 4 benzene derivatives (Figure 6C). Compared with Northeastern Chinese dajiang, Korean doenjang showed a higher content of phenol, p-cresol, 2-methoxy-4-vinylphenol, tetramethylpyrazine, butanoic acid, and ethyl benzoate. Notably, 4-ethyl-2-methoxyphenol was considered a differential component in the two types of soybean pastes, which is consistent with the results of Li et al. [1]. Sugars in soybeans can be utilized by heterotypicfermentative lactic acid bacteria to produce organic acids, such as lactic and acetic acids. Han et al. [12] discovered that the abundance of heterotypicfermentative lactic acid bacteria (such as *Tetragenococcus*, *Pediococcus*, *Wessella* and *Enterococcus*) was positively correlated with acetic acid content. Acetic acid was analyzed as a differential metabolite in Northeastern Chinese dajiang and Korean doenjang, which may be attributed to differences in the abundance of heterotypicfermentative lactic acid bacteria. In addition, phenylethyl alcohol not only enhances the aroma of the soybean pastes because of its rose and mellow aroma, but also has antibacterial, antiseptic, and disinfectant effects [49]. Indole and benzaldehyde affect the overall odor of the soybean pastes through their own fruity odors, whereas benzenacetaldehyde affects the overall flavor of the soybean pastes through its own flower odor [44]. Tetramethylpyrazine is believed to impart a nutty, chocolate, or baking aroma to the soybean pastes, which might be derived from Maillard reactions or produced by *Bacillus* spp. [50].

## 4. Conclusions

Dajiang and Doenjang are traditional fermented soybean pastes from Northeast China and Korea, respectively, boasting a long history of consumption. In our study, we examined the physicochemical and flavor attributes of these two soybean pastes, unearthing noteworthy distinctions between them. Through our analysis, we identified 19 volatile compounds that stand as differential volatile metabolites in dajiang and doenjang. These compounds likely play a pivotal role in generating the distinct flavors characterizing these two soybean paste varieties. In conclusion, our findings lay the groundwork for comprehending the flavor disparities between dajiang and doenjang, elucidating these distinctions through the lens of volatile metabolites. Functional and metabolic pathway analyses of microorganisms will be conducted in subsequent studies, which will facilitate the elucidation of the metabolic mechanisms of flavor formation in the two types of soybean pastes.

## Figures and Tables

**Table 1:** Information about soybean paste samples collected.

Sample	Raw Materials	Origin	Latitude (°)	longitude (°)
C1	Soybean, water, salt	Shenyang, Liaoning	123.41	41.80
C2	Soybean, water, salt	Shenyang, Liaoning	123.41	41.80
C3	Soybean, water, salt	Shenyang, Liaoning	123.41	41.80
C4	Soybean, water, salt	Shenyang, Liaoning	123.41	41.80
C5	Soybean, water, salt	Shenyang, Liaoning	123.48	41.85
C6	Soybean, water, salt	Shenyang, Liaoning	123.48	41.85
C7	Soybean, water, salt	Shenyang, Liaoning	123.48	41.85
K1	Soybean, water, salt	Yanji, Jilin	129.51	42.91
K2	Soybean, water, salt	Yanji, Jilin	129.51	42.91
K3	Soybean, water, salt	Yanji, Jilin	129.51	42.91
K4	Soybean, water, salt	Yanji, Jilin	129.51	42.91
K5	Soybean, water, salt	Yanji, Jilin	129.51	42.91
K6	Soybean, water, salt	Yanji, Jilin	129.51	42.91
K7	Soybean, water, salt	Yanji, Jilin	129.51	42.90

**Table 2:** Color properties of soybean paste samples.

Sample	L*	a*	b*
C1	37.3 ± 0.04 ^cd^	1.89 ± 0.05 ^h^	14.02 ± 0.10 ^e^
C2	33.63 ± 0.04 ^i^	2.92 ± 0.20 ^f^	12.25 ± 0.19 ^h^
C3	32.26 ± 0.04 ^j^	1.82 ± 0.08 ^h^	10.05 ± 0.11 ^i^
C4	37.7 ± 0.15 ^cd^	3.80 ± 0.27 ^d^	16.93 ± 0.37 ^a^
C5	40.34 ± 0.23 ^a^	3.42 ± 0.12 ^e^	17.08 ± 0.40 ^a^
C6	35.43 ± 0.16 ^h^	2.97 ± 0.14 ^f^	13.99 ± 0.43 ^e^
C7	37.35 ± 0.14 ^de^	2.57 ± 0.06 ^g^	14.42 ± 0.18 ^de^
K1	39.50 ± 0.45 ^b^	5.39 ± 0.09 ^a^	16.70 ± 0.18 ^a^
K2	37.83 ± 0.63 ^cd^	4.70 ± 0.06 ^b^	15.01 ± 0.21 ^bc^
K3	36.86 ± 0.28 ^de^	4.44 ± 0.05 ^c^	14.80 ± 0.38 ^cd^
K4	36.63 ± 0.38 ^f^	4.01 ± 0.09 ^d^	13.41 ± 0.32 ^f^
K5	38.23 ± 0.16 ^c^	4.76 ± 0.03 ^b^	15.31 ± 0.03 ^b^
K6	36.34 ± 0.56 ^fg^	3.79 ± 0.12 ^d^	12.88 ± 0.09 ^g^
K7	35.83 ± 0.26 ^gh^	3.79 ± 0.07 ^d^	12.36 ± 0.23 ^h^

Different letters are significantly different at *p* < 0.05.

## Data Availability

The data presented in this study are available in the article.

## Supplementary Materials

The following supporting information can be downloaded at: https://www.mdpi.com/article/10.3390/foods12173294/s1.
